# Efficacy and safety of low-dose rituximab in the treatment of myasthenia gravis: a systemic review and meta-analysis

**DOI:** 10.3389/fneur.2024.1439899

**Published:** 2024-09-25

**Authors:** Xishuai Yang, Wei Zhang, Junhong Guo, Chunlin Ma, Bingxia Li

**Affiliations:** ^1^Department of Neurology, Changzhi People's Hospital, Changzhi, China; ^2^Department of Neurology, The First School of Shanxi Medical University, Taiyuan, China; ^3^Department of Neurology, The First Hospital of Shanxi Medical University, Taiyuan, China

**Keywords:** low-dose, RTX, myasthenia gravis, acetylcholine receptor, muscle-specific kinase

## Abstract

**Background:**

Rituximab (RTX) is a monoclonal antibody that has been increasingly used in the treatment of myasthenia gravis (MG). In most studies, the therapeutic protocol of RTX has been similar to that adopted for B cell lymphoma, with an increasing number of studies aimed at exploring the efficacy of low-dose RTX in MG. However, the beneficial effects of low-dose RTX in MG remain a subject of critical debate.

**Methods:**

This study was conducted following the PRISMA (Preferred Reporting Items for Systematic Review and Meta-Analysis) guidelines. Two reviewers (Xishuai Yang and Bingxia Li) independently conducted searches across multiple databases, including PubMed, MEDLINE, EMBASE, Web of Science, Cochrane Library, and China National Knowledge Infrastructure (CNKI). A meta-analysis, utilizing representative forest plots, was performed to assess “Improved clinical status” and changes in the Quantitative Myasthenia Gravis (QMG) score before and after treatment.

**Results:**

A total of 17 studies involving 292 patients were included in the meta-analysis. A noticeable improvement in clinical status was observed in 91% of patients at the final follow-up after therapy (95% CI: 84–96%, *P* < 0.001). The QMG score showed a significant reduction following the treatment, with a standardized mean difference (SMD) of −1.69 (95% CI: −2.21 to −1.16, *Z* = 6.29, *P* < 0.001). In the acetylcholine receptor antibody-positive myasthenia gravis (AChR-MG) group, 90% of patients achieved improved clinical status (95% CI: 80–97%, *P* < 0.001) and the QMG score significantly decreased after low-dose RTX treatment, with an SMD of −1.51 (95% CI: −0.80 to −2.21, *Z* = 4.50, *P* < 0.001). In the muscle-specific kinase antibody-positive myasthenia gravis (MuSK-MG) group, 97% of patients achieved improved clinical status (95% CI: 89–100%, *P* < 0.001). The QMG score also significantly decreased following low-dose RTX treatment, with an SMD of −2.31 (95% CI: −2.99 to −1.62, *Z* = 6.60, *P* < 0.001). Adverse effects were reported in 29 out of 207 patients (14%, including infusion reactions in 22 patients (10.1%), infections in three patients (1.45%), cytopenia in two patients (0.96%), eosinophilia in one patient (0.48%), and hemiplegia in one patient (0.48%). Additionally, one patient (0.48%) succumbed to complications from invasive thymoma.

**Conclusion:**

Our meta-analysis shows that low-dose RTX is both effective and safe for treating MG.

**Systematic Review Registration:**

PROSPERO, identifier: CRD42024509951.

## 1 Introduction

Myasthenia gravis is an autoimmune disease characterized by the presence of antibodies at the neuromuscular junction. In 85–90% of cases, these autoantibodies target acetylcholine receptors, while other cases involve antibodies against muscle-specific kinase, lipoprotein-related protein 4, or agrin (1, 2).

Although MG is usually treated effectively with acetylcholinesterase inhibitors, corticosteroids, or corticosteroid-sparing agents, such as azathioprine and mycophenolate mofetil, 10–15% of patients have difficult-to-control disease, commonly referred to as treatment-refractory MG (3). Rituximab (RTX), a monoclonal antibody that targets the CD20 antigen found in 95% of mature B cells, acts by initiating complement-dependent cytolysis or antibody-dependent cell-mediated cytotoxicity (4).

The effectiveness of RTX in treating MG was first reported in 2000 (5). Over the past two decades, research studies have suggested that RTX is effective for refractory MG (6–9). A systematic review of retrospective reports on RTX responsiveness in MG patients indicated that RTX appears to be particularly effective for MG patients, especially those with MuSK antibodies (10).

In most studies, the therapeutic protocol of RTX has been similar to that used for B cell lymphoma, involving induction therapy with either 4 weekly doses of 375 mg/sm^2^ or 2 doses of 1,000 mg on days 1 and 15, followed by reinfusion every 6 months (8, 11–14). However, RTX is expensive; the average cost of one treatment cycle in a previous study was estimated to be approximately $30,000 (15).

Furthermore, high-dose RTX has been associated with adverse reactions, including myocardial infarction, spondylodiscitis, neutropenia, agranulocytosis, and diverticulitis, along with two cases of progressive multifocal leukoencephalopathy (13, 16, 17). To reduce both the cost and potential risks associated with high-dose RTX, some studies have suggested that low-dose RTX could improve clinical symptoms in MG patients (18–21).

To achieve this objective, we conducted the first meta-analysis aimed at providing robust evidence on the effectiveness and safety of low-dose rituximab in the treatment of myasthenia gravis.

## 2 Methods

### 2.1 Study selection and data collection

Our meta-analysis, conducted in accordance with PRISMA guidelines, aimed to evaluate the role of low-dose rituximab (RTX) as a treatment for myasthenia gravis (MG). The PRISMA checklist and flow diagram were used to guide the study selection process. Two reviewers, Xishuai Yang and Bingxia Li, independently searched databases such as PubMed, MEDLINE, EMBASE, Web of Science, Cochrane Library, and China National Knowledge Infrastructure (CNKI) for studies published between January 2000 and April 2024. The search terms used were “myasthenia gravis” and “low-dose rituximab.”

**Figure 1:**
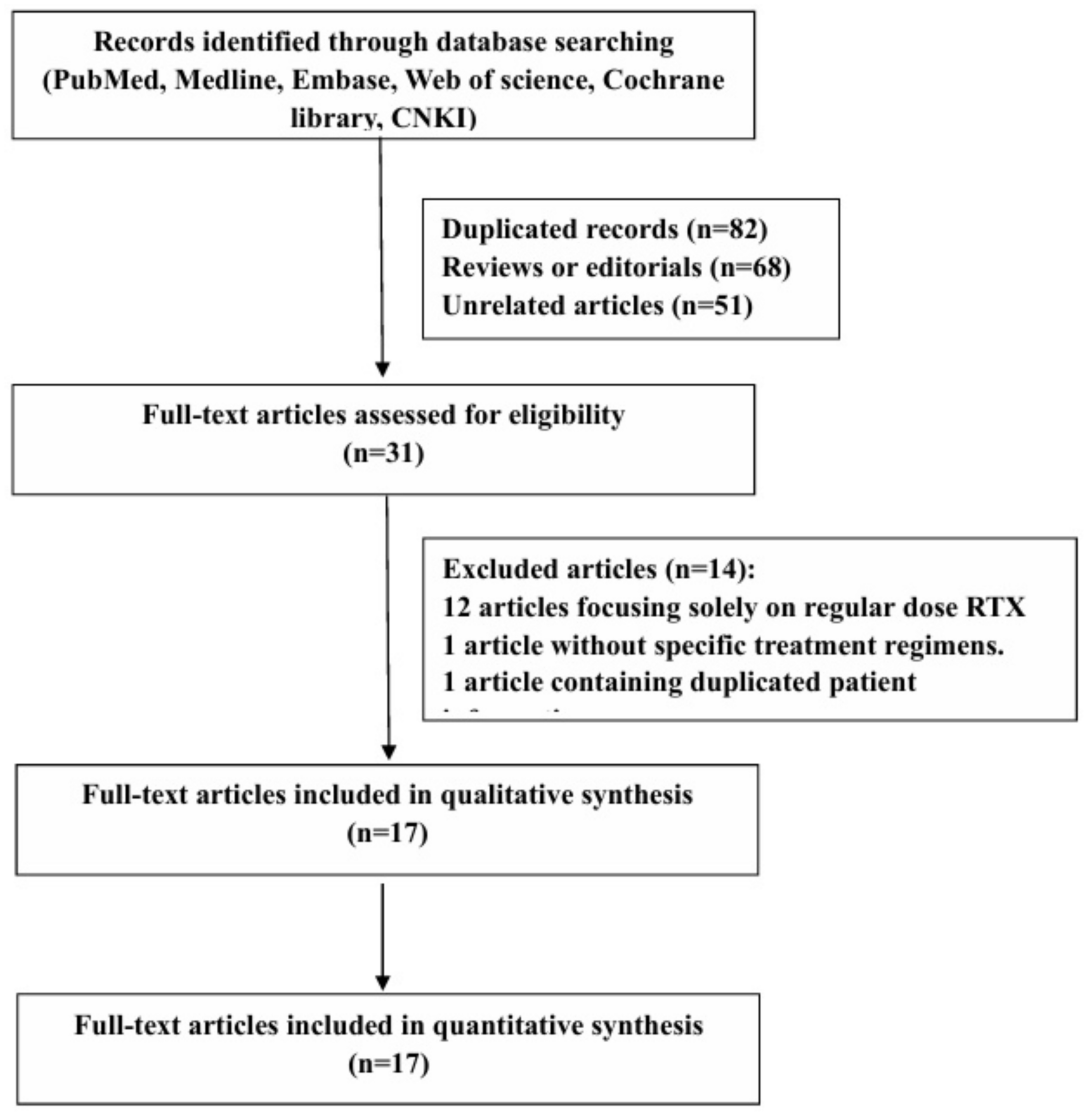
Study selection algorithm.

The initial search yielded 232 articles, of which 82 were duplicates, 68 were reviews, and 51 were unrelated studies. The titles and abstracts were screened, and 31 full texts of the relevant articles were reviewed based on inclusion and exclusion criteria. A flow chart of the search strategy is shown in Figure 1. Ultimately, only one randomized controlled trial and 16 observational studies were included.

Inclusion criteria:

Study type(s): Prospective or retrospective studies published in English or Chinese.

Study participant(s): Individuals of any age, gender, or nationality treated with low-dose rituximab for MG.

Study intervention(s): Low-dose rituximab, administered according to various regimens, was assessed as a treatment for MG. In this study, “low dose” was defined as fewer than 4 weekly doses of 375 mg/m^2^ or a dosing regimen of <1,000 mg repeated at a 2-week interval, with or without subsequent maintenance therapy.

Objective outcome(s): The primary outcome was the proportion of patients achieving improved clinical status, as measured by the Myasthenia Gravis Foundation of America post-intervention status (MGFA-PIS) categories of minimal manifestation status (MMS), pharmacologic remission (PR), complete stable remission (CSR), or improved (I) at the final follow-up. The secondary outcomes included changes in QMG scores from baseline to final follow-up.

Studies were included if they focused on the above-mentioned primary or secondary outcomes.

Exclusion criteria:

Articles focused solely on regular-dose RTX.

Articles without specific treatment regimens.

Articles with duplicate patient information were combined for comprehensive data but treated as a single case.

Extracted data included study design, participant characteristics, and outcome measures. For each study, the following information was retrieved: the first author, study design, year of publication, language, number of patients receiving low-dose rituximab, MG subtype, mean onset age, mean enrolled age, proportion of women, antibody type, follow-up duration, minimum follow-up time, mean disease duration, Myasthenia Gravis Foundation of America post-intervention status (MGFA-PIS), and QMG scores pre- and post-RTX therapy.

Chunlin Ma extracted the data using a standardized form, which was then reviewed by Wei Zhang and Junhong Guo. Discrepancies were resolved through discussion and consensus. The study protocol was prospectively registered with PROSPERO.

### 2.2 Efficacy and safety measures

The primary efficacy outcome measure evaluated in this meta-analysis was the proportion of patients achieving “improved clinical status”. “Improved clinical status” was characterized as achieving complete stable remission (CSR), pharmacologic remission (PR), minimal manifestations (MM), or improved (I), as classified by the Myasthenia Gravis Foundation of America-post-intervention status (MGFA-PIS).

The secondary outcomes included changes in the QMG score from baseline to final follow-up.

Safety outcomes were extracted from all the studies included in the meta-analysis. These outcomes included the proportion of adverse events, deaths, and withdrawals due to toxicity and adverse events.

### 2.3 Statistical analysis

The primary outcome was expressed as a transformed effect size and analyzed using a random-effects meta-analysis model.

The effect size of secondary outcomes was calculated as the standard mean difference (SMD), with a 95% confidence estimated using an appropriate model. The *I*^2^ test was employed to assess the presence of heterogeneity. A fixed-effects model was selected if the *I*^2^ value was <50%; otherwise, a random-effects model was used.

Forest plots were generated to provide an overview of results of the included studies and the combined effects. The potential for publication bias was evaluated through visual inspection of the funnel plot and Egger's test. The trim-and-fill method was used to calculate the adjusted effect size, accounting for potential publication bias. All analyses were performed using Stata/MP15 software (College Station, Texas 77845 USA).

## 3 Results

### 3.1 Study characteristics

A total of 17 studies were identified and included by screening the literature in the meta-analysis. The combined datasets from these 17 studies included a total of 292 MG patients treated with low-dose RTX. Among these patients, 208 (71.2%) were positive for AChR antibodies (AChR-Ab+), 70 (24%) were positive for MuSK antibodies (MuSK-Ab+), 1 (0.34%) was positive for LRP antibodies (LRP-Ab+), and 13 (4.45%) were antibody-negative.

The mean age at the time of the first dose was 51.98 ± 19.20 years. The mean disease duration was 55.06 ± 76.22 months, and the mean follow-up time was 23.30 ± 19.67 months. The doses of RTX mentioned in these studies varied and were lower than the standard protocol. These details are shown in Table 1.

**Table 1:** The clinical features and RTX regimen in patients with MG receiving low-dose RTX therapy.

**References**	**Study design**	**Age at first dose (years), mean (SD)**	**Total (female)**	**Antibody subtype**	**MG subtype**	**Disease duration (months), mean (SD)**	**Follow-up time (months), mean (SD), minimum**	**RTX regimen**
Lu et al. (27)	Prospective	30.6(29.6)^*^	12(10)	AchR+	Refractory generalized	59.6(37.7)	18(0), 18	Induction treatment: 600 mg Maintenance treatment:600 mg every 6 months
Brauner et al. (20)	Retrospective	60(18)	72(31)	AchR+	Generalized	67(95)	40(19), NA	Induction treatment: 1,000 mg in 3 patients, 500 mg in 57 patients, 100 mg for 12 patients. Maintenance treatment: 500 mg in 69 patients, 100 mg every 6 months in 3 patients
Piehl et al. (19)	Randomized clinical trial	67.4(13.4)	25(7)	AchR+	New onset generalized	4.47(3.05)	6(0), 6	A single dose of 500 mg RTX
Zhou et al. (28)	Retrospective	40(13.85)	12(11)	Musk+	Generalized	21.42(22.11)	6(0), 6	100 mg (day1) and 500 mg (day2)
Zhao et al. (29)	Retrospective	NA	8(6)	Musk+	Generalized	41.38(63.19)	6(0), 6	Induction treatment: 100 mg weekly for 3 weeks. Maintenance treatment: 100 mg if CD19+ B cell > 1% or CD19+CD27+ memory B cells > 0.05% of PBMCS
Meng et al. (30)	Retrospective	44.9(9.7)^*^	8(8)	Musk+	Generalized	10.75(12.69)	22(8.55), 8	375 mg/m^2^ for 1 or 2 infusions
Yang et al. (21)	Retrospective	50.56(13.51)	9(8)	Musk+	Generalized	24.22(14.76)	13(6.4), 3	Induction treatment: 500 mg Maintenance treatment: 500 mg every 6 months or 1 year
Blum et al. (31)	Retrospective	50.08(18.72)	13(9)	Musk+	Generalized	10.08(13)	11.85(6.56), 4	500 mg weekly for 2 weeks in 12 patients, 500 mg in 1 patient
Choi et al. (32)	Retrospective	50.53(15.56)	17(11)	9AchR+, 6Musk+, 2Neg	Refractory generalized	122.2(88.3)	24.47 (11.29), 7	Induction treatment: 375 mg/m^2^ twice with a 2-week interval Maintenance treatment:375 mg/m^2^ once if B cell repopulates or relapse
Heckmann (33)	Retrospective	36.38(15.18)	17(16)	5musk, 10AchR+, 2Neg	Refractory generalized	134.2(108.5)	19.12(12.81), 6	A single dose of 375 mg/m^2^
Zhong et al. (34)	Prospective	30.8(13.5)	12(10)	AchR+	Refractory generalized	54.83(40.83)	6(0), 6	A single dose of 600 mg
Li et al. (35)	Retrospective	NA	19(12)	AchR+	New onset generalized	NA	51.55(11.32), 30.3	100 mg for 1 day, 2 days, 3 days, or 4 days if CD19+Bcells were (0.5%-1.5%), (1.5%-5%), (5%-15%), or (>15%) of PBMCS
Castiglione et al. (36)	Retrospective	NA	9(NA)	5AchR+, 4Musk+	Generalized	NA	24(0), 24	375 mg/m^2^ twice with a 2-week interval in 7 patients, 500 mg weekly for 3 weeks in 2 patients
Du et al. (37)	Retrospective	58.92(10.01)	13(7)	AchR+	New onset generalized	2.85(2.07)	25(11.21), 12	Induction treatment:100 mg no more than 3 weeks, Maintenance treatment:100 mg based on circulating B-cell repopulation every 3 months
Jing et al. (18)	Prospective	34.4(13.1)	15(14)	13AchR+, 1Musk+, 1 Neg	Refractory generalized	57.3(32.8)	6(0), 6	100 mg (day 1) and 500 mg (day2)
Ren et al. (38)	Retrospective	48.45(16.29)	22(18)	9AchR+, 4Musk+, 1LRP4, 8Neg	Refractory generalized	66 range(45-144)	Median 48.5 range(23-84)	Induction treatment: 100 mg in 12 patients, 200 mg in 2 patients, 500 mg in 7 patients, 100 mg (day1)and 500 mg (day 2) in 1 patient. Maintenance treatment: 500 mg in 17 patients, 100-200 mg in 5 patients if CD20+B-cell %>1% of PBMCS
Brauner et al. (20)	Prospective	53.44(12.77)	9(5)	AchR+	Generalized	NA	6(0), 6	100 mg weekly for 4 weeks in 6 patients, 100 mg in 3 patients

NA, not available.

* Age at onset.

### 3.2 Efficacy

#### 3.2.1 Efficacy on MG patients

##### 3.2.1.1 Proportion of patients achieving improved clinical status

**Figure 2:**
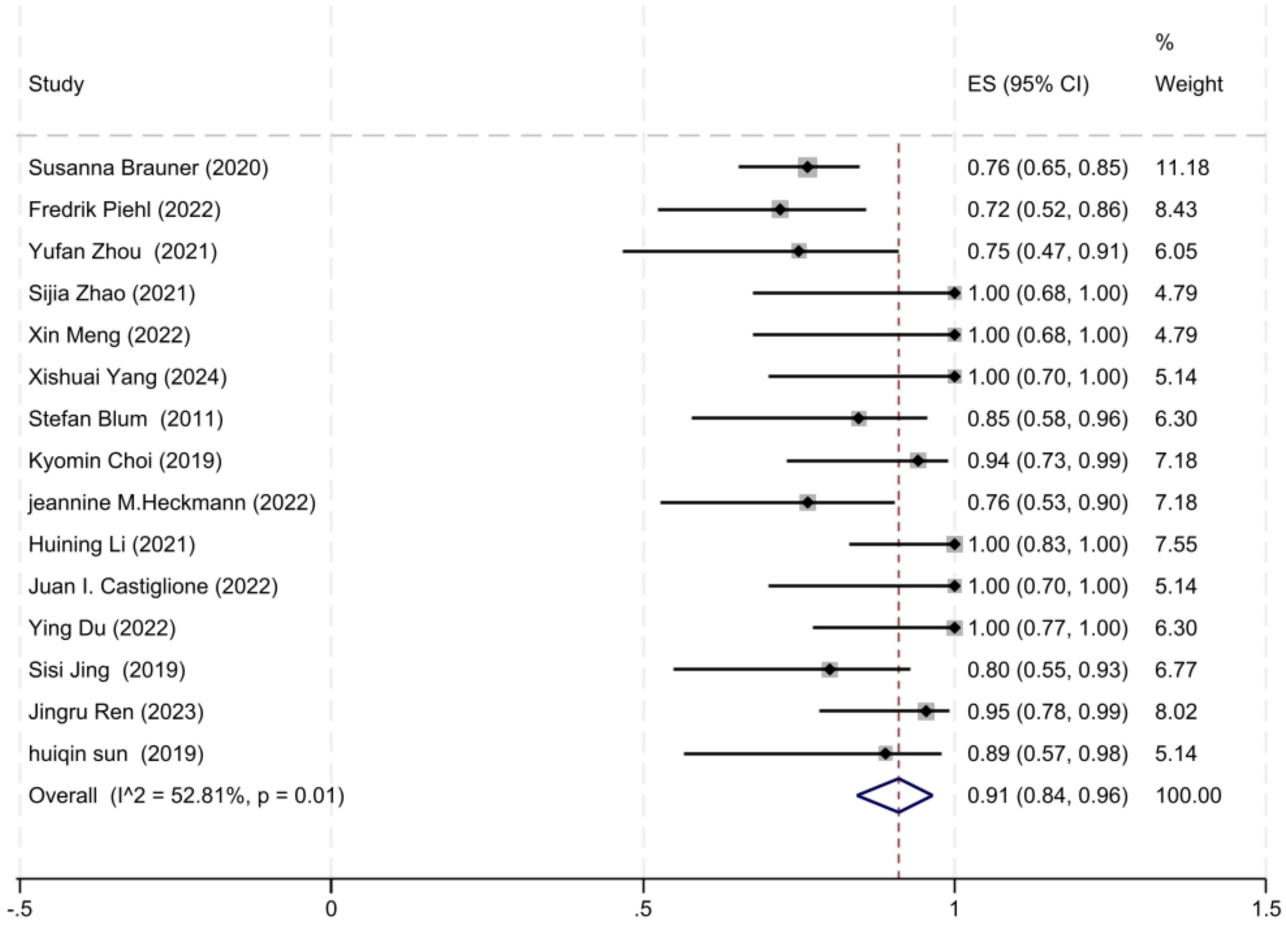
The forest plot illustrating the improvement rate associated with low-dose RTX therapy.

A total of 15 out of 17 studies reported the number of patients who achieved improved clinical status at the final follow-up. The degree of heterogeneity among these studies was moderate (*I*^2^ = 52.81%, *P* = 0.01), prompting the use of a random-effects model for the meta-analysis. Our meta-analysis revealed that 91% of patients (95% CI: 84–96%) exhibited improved clinical status at the final follow-up after receiving low-dose RTX therapy, which was statistically significant (*z* = 23.32, *P* < 0.001). The forest plot illustrating the improvement rate associated with low-dose RTX therapy is shown in Figure 2.

##### 3.2.1.2 Efficacy in reducing QMG scores

**Figure 3:**
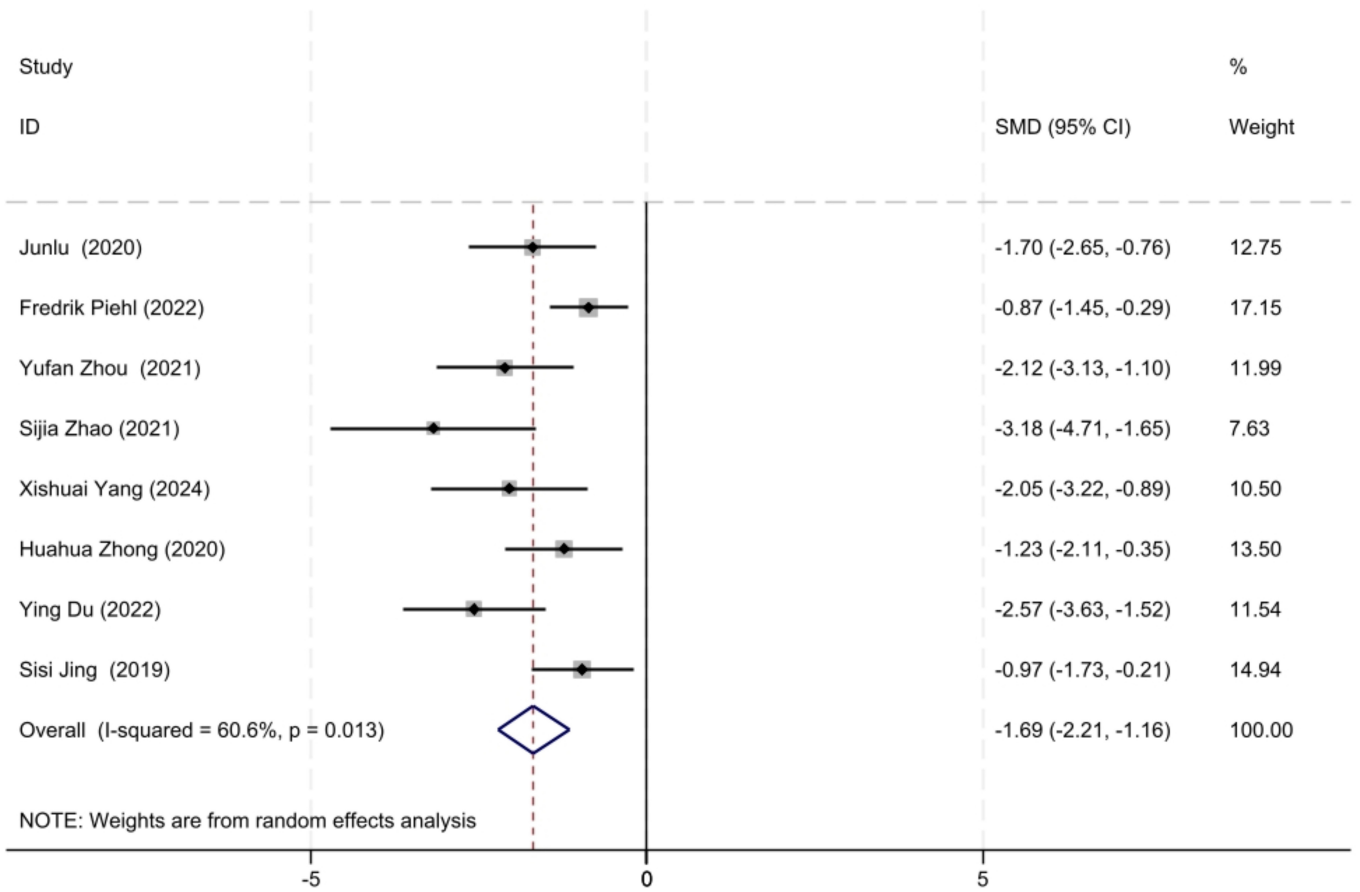
The forest plot illustrating the reduction in QMG scores following low-dose RTX treatment.

Baseline QMG scores and QMG scores at the final follow-up were reported in eight out of the 17 studies. Given the moderate heterogeneity among the studies (*I*^2^ = 60.6%, *P* = 0.013), a random-effects model was utilized for the meta-analysis. Our meta-analysis revealed that the initiation of low-dose RTX significantly reduced the QMG scores at follow-up, with an SMD of −1.69 (95% CI: −2.21 to −1.16), which was statistically significant (*Z* = 6.29, *P* < 0.001). The forest plot depicting the reduction in QMG scores following low-dose RTX treatment is shown in Figure 3.

#### 3.2.2 Efficacy on AChR-MG patients

##### 3.2.2.1 Proportion of patients achieving improved clinical status

**Figure 4:**
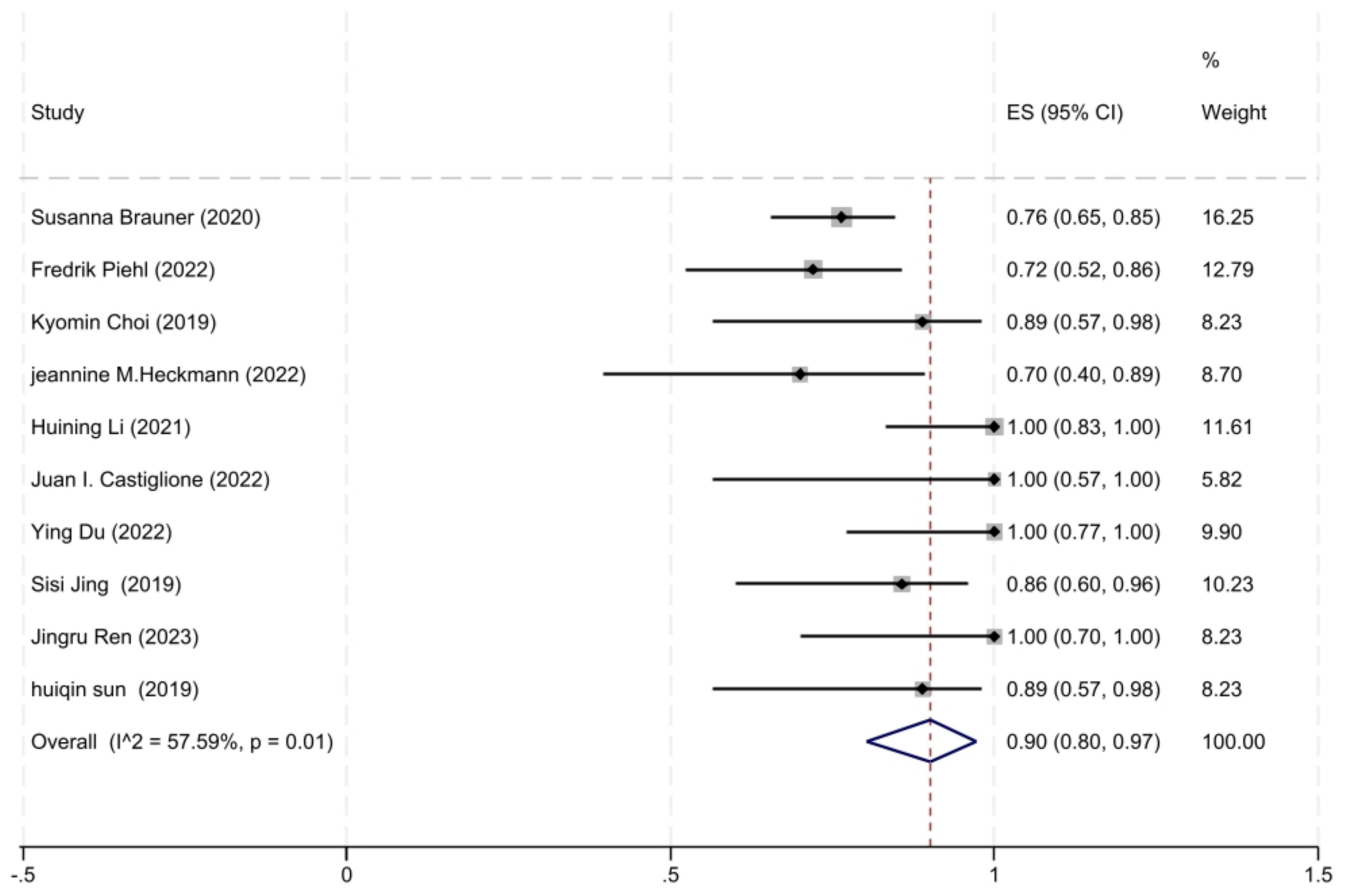
The forest plot illustrating the improvement rate after low-dose RTX therapy in AChR-MG patients.

A total of 10 studies involving AChR-MG patients with MGFA-PIS data were included in the analysis. The heterogeneity between studies, as assessed by the *I*^2^ test, was 57.59% (*P* = 0.01), leading us to employ a random-effects model for the meta-analysis. The analysis revealed that 90% of AChR-MG patients (95% CI: 80–97%) attained improved clinical status, a result that was statistically significant (*z* = 17.15, *P* < 0.001). The forest plot depicting the improvement rate in AChR-MG patients treated with low-dose RTX is shown in Figure 4.

##### 3.2.2.2 Efficacy of QMG score reduction

**Figure 5:**
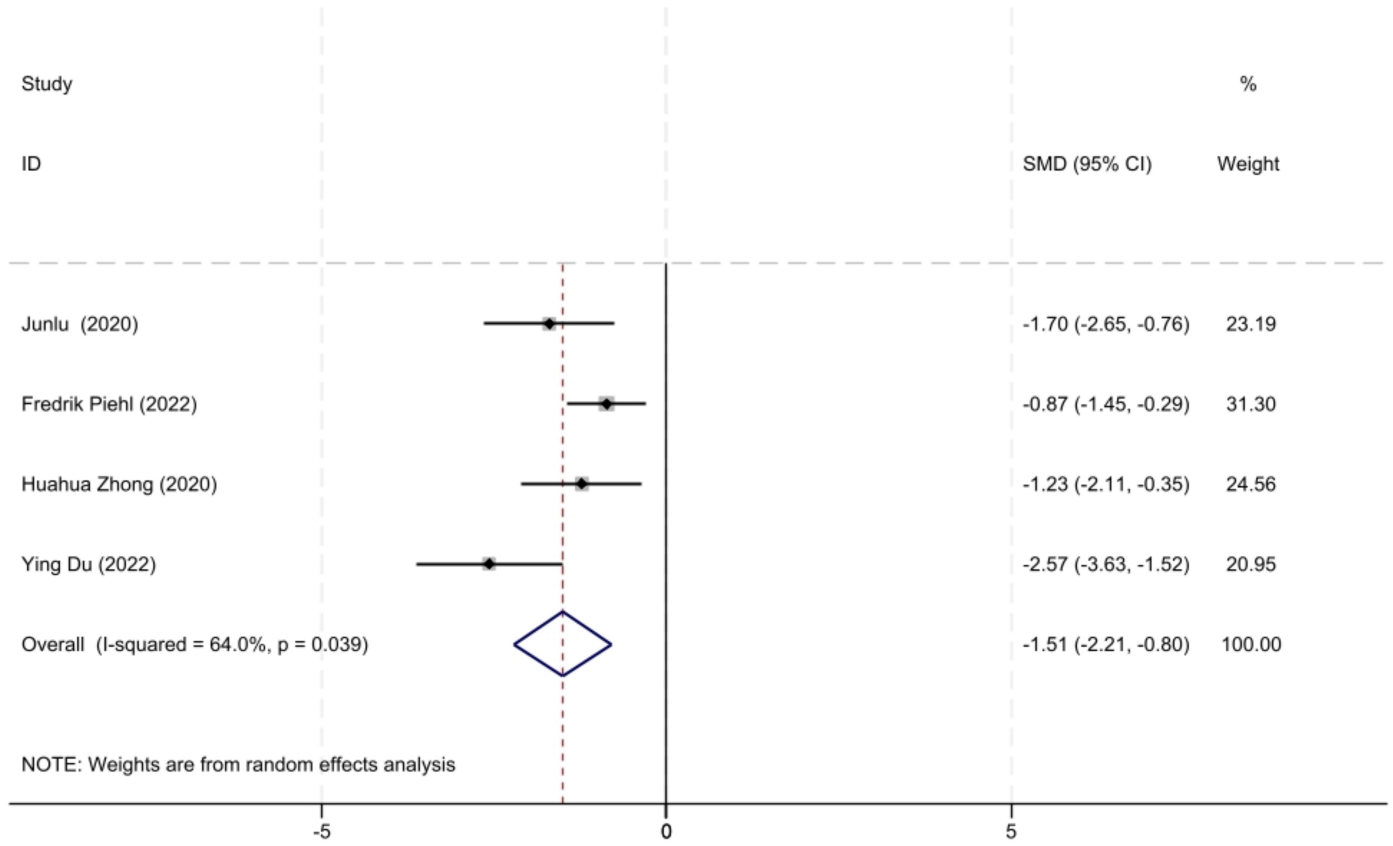
The forest plot illustrating the reduction in QMG scores after low-dose RTX therapy in AChR-MG patients.

A total of four studies involving AChR-MG patients with QMG data were included in the analysis. Considering the moderate heterogeneity among these studies (*I*^2^ = 64.0%, *P* = 0.039), a random-effects model was utilized for the meta-analysis. The results demonstrated that low-dose RTX significantly reduced QMG scores at follow-up, with an SMD of −1.51 (95% CI: −2.21 to −0.80, *Z* = 4.5, *P* < 0.001), which was statistically significant. The forest plot illustrating the reduction in the QMG scores following low-dose RTX treatment in AChR-MG patients is shown in Figure 5.

#### 3.2.3 Efficacy on MuSK-MG patients

##### 3.2.3.1 Proportion of patients achieving improved clinical status

**Figure 6:**
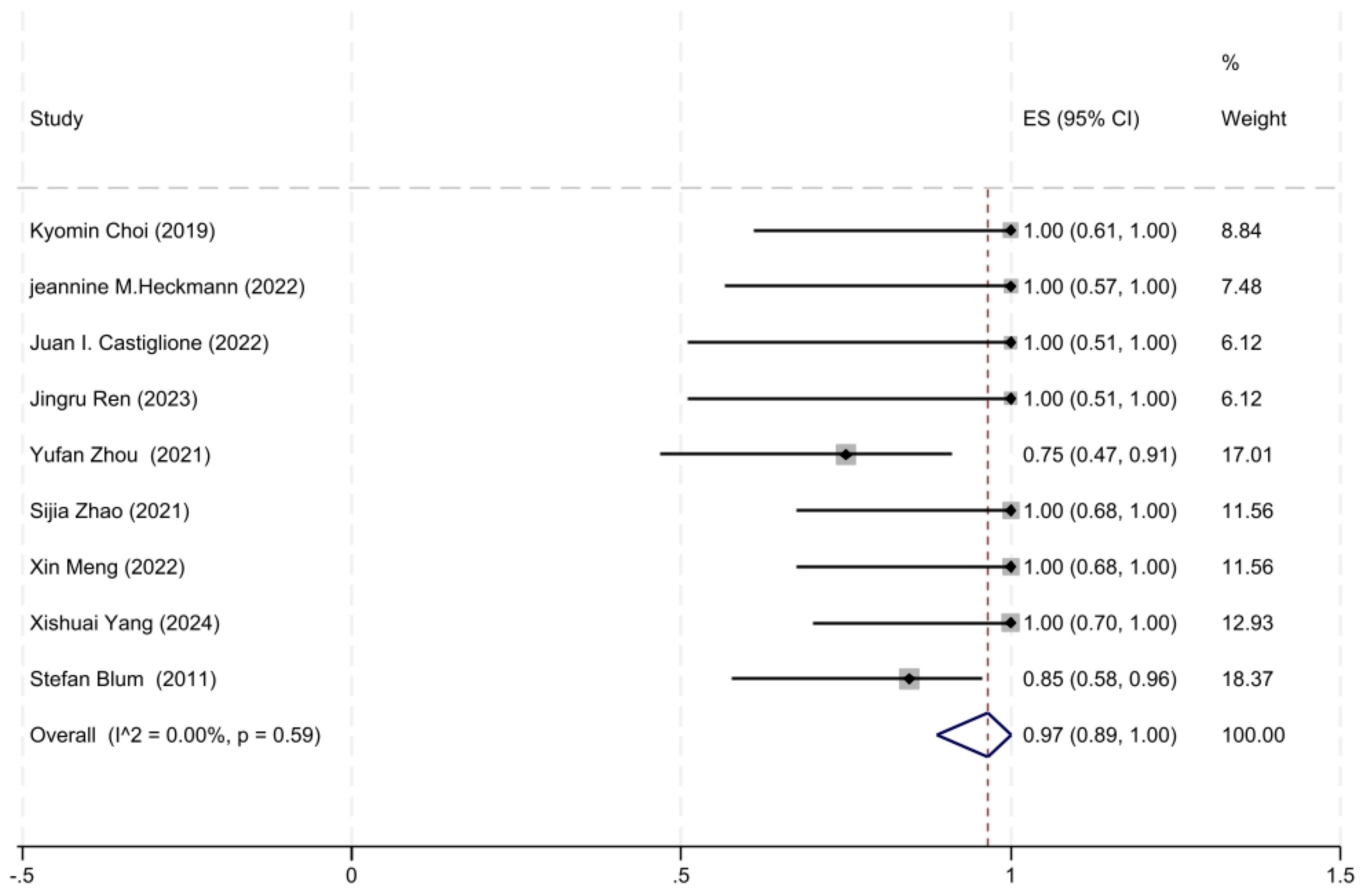
The forest plot illustrating the improvement rate after low-dose RTX therapy in MuSK-MG patients.

A total of nice studies involving MuSK-MG patients with MGFA-PIS data were included in the analysis. Given the moderate heterogeneity among these studies (*I*^2^ = 0.00%, *P* = 0.59), a fixed-effects model was used for the meta-analysis. The analysis revealed that 97% of MuSK-MG patients (95% CI: 89–100%) reached improved clinical status (95% CI: 89–100%), which was statistically significant (*z* = 18.69, *P* < 0.001). The forest plot depicting the improvement rate in MuSK-MG patients treated with low-dose RTX is shown in Figure 6.

##### 3.2.3.2 Efficacy of QMG scores reduction

**Figure 7:**
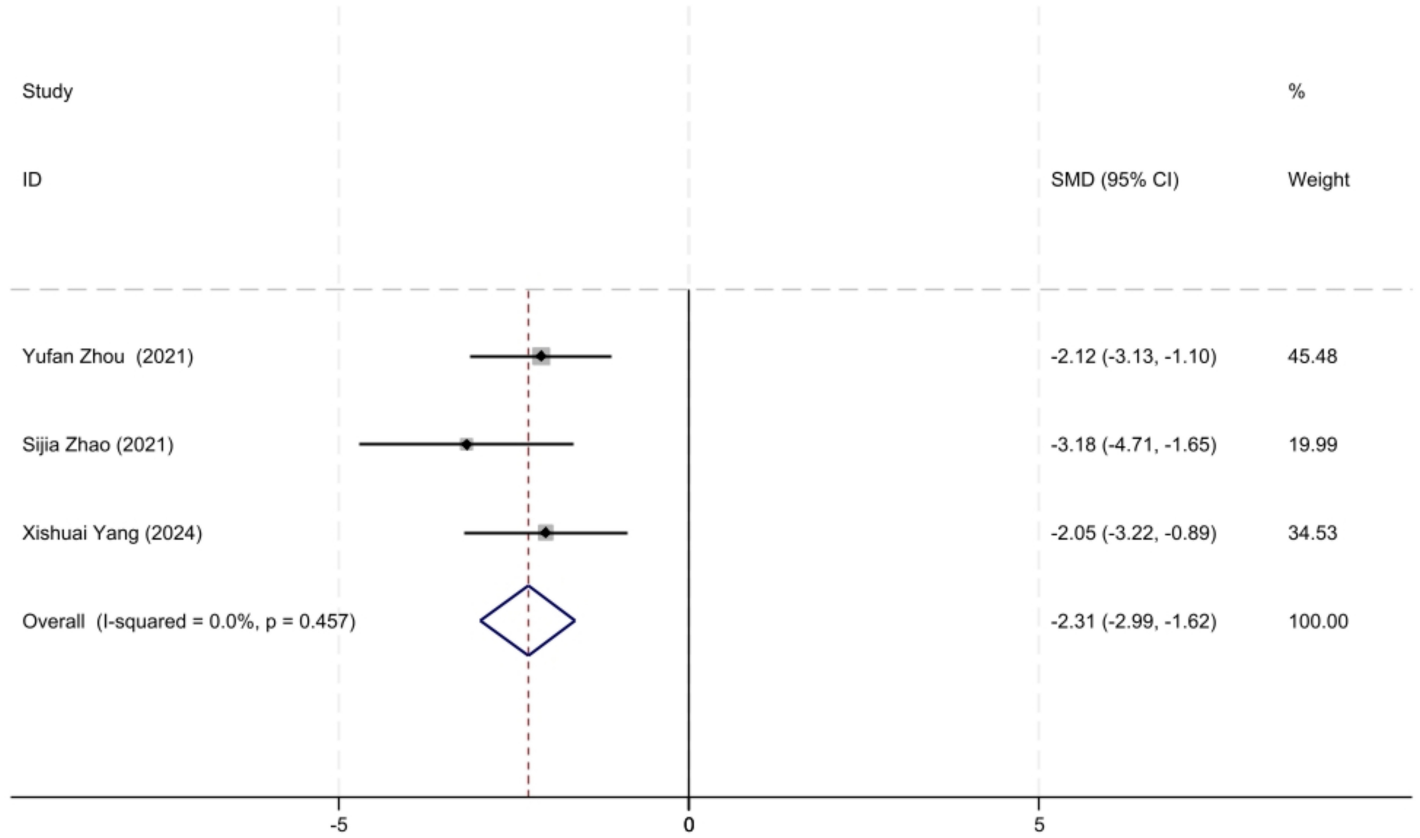
The forest plot illustrating the reduction in QMG scores in MuSK-MG patients following low-dose RTX therapy.

A total of three studies involving MuSK-MG patients with QMG data were included in the analysis. The lack of heterogeneity among studies, as assessed by the *I*^2^ test (*P* = 0.457), prompted the use of a fixed-effects model for the meta-analysis. The meta-analysis showed that the initiation of low-dose RTX significantly reduced QMG scores at follow-up in MuSK-MG patients, with an SMD of −2.31 (95% CI: −2.99 to −1.62), a result that was statistically significant (*Z* = 6.60, *P* < 0.001). A forest plot illustrating the reduction in QMG scores in MuSK-MG patients following low-dose RTX treatment is shown in Figure 7.

### 3.3 Safety

A total of 10 studies reported adverse events associated with low-dose RTX treatment. During the follow-up period, 29 out of 207 patients (14%) experienced adverse events. These adverse effects varied, with infusion reactions occurring in 22 patients (10.1%) and infections—specifically, one case of pneumonia and two cases of oral herpes zoster—in 3 patients (1.45%).

Additionally, cytopenia was observed in two patients (0.96%), eosinophilia in one patient (0.48%), and hemiplegia in one patient (0.48%). Two patients (0.96%) discontinued rituximab treatment owing to adverse effects: one patient experienced an allergic infusion reaction during the second infusion (subsequently treated with atumumab but eventually returned to rituximab), and another had recurrent episodes of pneumonia. Furthermore, one patient (0.48%) passed away due to complications related to invasive thymoma.

### 3.4 Publication bias

**Figure 8:**
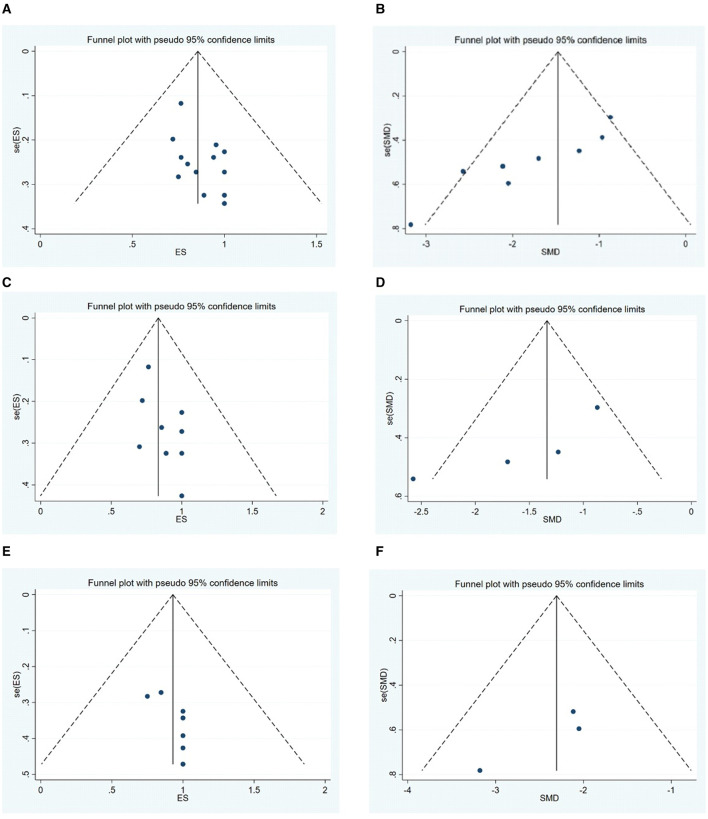
**(A–F)** Funnel plots illustrating publication bias for the outcomes.

Publication bias for the outcomes, including the MGFA-PIS improvement rate and QMG scores, was assessed through visual inspection of funnel plots and the application of Egger's Test. The funnel plots are illustrated in Figure 8. The findings suggest a potential publication bias for the MGFA-PIS improvement rate, QMG scores in all MG patients, and the MGFA-PIS improvement rate in the MuSK-MG group, as shown in Table 2 (*P* < 0.05).

**Table 2:** The results for publication bias with Egger's test.

**Antibody type group**	**Outcome**	**Coef**.	**Std. err**.	***t***	***P***
ALL	MGFA-PIS	0.906	0.318	8.81	0.014
	QMG	−5.145	0.85	−6.05	0.001
AchR-MG	MGFA-PIS	0.739	0.356	2.08	0.071
	QMG	−5.431	1.849	−2.94	0.099
Musk-MG	MGFA-PIS	1.063	0.409	2.6	0.035
	QMG	−4.066	1.812	−2.24	0.267

Trim-and-fill tests were conducted to address the possibility of publication bias. The results for the MGFA-PIS improvement rate and QMG scores using the trim-and-fill test are shown in Table 3.

**Table 3:** The results for publication bias using the trim-and-fill test.

**Antibody type group**	**Outcome**	***n***	**Before trim and fill**				**After trim and fill**			
			**Pooled estimate**	**Lower limit**	**Upper limit**	***P***	**Pooled estimate**	**Lower limit**	**Upper limit**	***P***
All	MGFA-PIS	4	0.856	0.738	0.974	0.000	0.807	0.702	0.913	0.000
	QMG	0	−1.686	−2.211	−1.16	0.000	−1.686	−2.21	−1.16	0.000
Musk-MG	MGFA-PIS	3	0.929	0.701	1.158	0.000	0.902	0.7	1.104	0.000

*n*, studies trimmed or filled.

No trimming was performed for the QMG scores. The results before and after winsorization remained statistically significant, indicating that the primary conclusions are robust despite the data processing step. Overall, the trim-and-fill test suggested the stability of the results for each outcome.

## 4 Discussion

Our meta-analysis indicated that low-dose RTX is a promising treatment for MG patients. After an average follow-up of 23.30 months, 91% of MG patients showed improved clinical status following low-dose rituximab therapy.

Although MG subtypes defined by autoantibody specificity may appear clinically similar, their underlying immunopathology is remarkably distinct (22). Several case reports and clinical series have shown that RTX is particularly effective in treating MuSK-MG, often yielding better outcomes than AChR-MG (12–14).

In our analysis, 90% of AChR-MG and MuSK-MG patients achieved improved clinical status. These outcomes are comparable to those observed with the standard RTX protocol. In a previous meta-analysis, the response rate of MG patients to RTX was 83.9%, with MuSK-MG patients showing a higher response rate (88.8%) than AChR-MG patients (80.4%) (23). Another meta-analysis revealed that the proportion of patients achieving MGFA-PIS improvement in the lower-dose RTX group (77.1%) was slightly higher than that in the standard protocol group (76.8%) (10).

These studies suggest that both low-dose and standard-dose RTX are effective in treating MG, with low-dose RTX being as effective as standard protocol RTX. The higher ratio in our meta-analysis compared to previous studies may be because we included pharmacologic remission (PR) as part of the improved clinical status classified by MGFA-PIS, whereas the previous study did not (24).

Our meta-analysis demonstrated that low-dose RTX effectively reduced QMG scores in all MG patients (SMD = −1.69, *P* < 0.001), especially in those with MuSK antibodies (SMD = −2.31, *P* < 0.001). MuSK-MG antibodies are primarily of the IgG4 class, unlike the IgG1 antibodies found in AChR-MG (25). The remarkable efficacy of RTX in MuSK-MG patients may be due to its selective depletion of cells that produce IgG4 antibodies specific to MuSK (26).

In this meta-analysis, the incidence of mild adverse effects during the follow-up period was 14% (29/207) among patients, which is lower than the 26.1% reported for patients receiving standard-dose rituximab in a previous meta-analysis (24). Additionally, low-dose rituximab was associated with a reduced rate of drug discontinuation due to adverse events (0.96%), compared to 46% observed with conventional immunotherapy in a previous study (20).

The meta-analysis had several limitations. First, the sample size was relatively small, with most studies focusing on AChR-Ab+ patients, while the number of MuSK-Ab+ patients was very limited, and there were no data on patients with LRP4 or agrin antibodies. Second, the RTX regimens and follow-up periods varied across studies.

Third, observational studies often overestimate treatment effects compared to randomized trials, which may lead to an overestimation of results. These factors, among others, could contribute to heterogeneity in the study. However, despite heterogeneity and publication bias, the results before and after Winsorization remained statistically significant, indicating that the primary conclusions are robust. To improve research quality, conducting a multi-center randomized controlled trial with a standardized low-dose RTX regimen in a larger MG patient population is recommended, which would allow for further assessment of the efficacy and safety of low-dose RTX.

## 5 Conclusion

Our meta-analysis indicated that low-dose RTX is effective for both AChR-MG and MuSK-MG. Furthermore, the majority of MG patients responded well to RTX. To further assess the efficacy and safety of RTX, we recommend conducting a multi-center randomized controlled clinical trial with a low-dose RTX regimen in a larger population of MG patients.

## Data Availability

The original contributions presented in the study are included in the article/supplementary material, further inquiries can be directed to the corresponding author.
